# Transthyretin Amyloid Cardiomyopathy: Current Diagnostic Approach and Risk Stratification with Multimodality Imaging

**DOI:** 10.3390/jcm14062014

**Published:** 2025-03-16

**Authors:** Georgios Tziomalos, Thomas Zegkos, Eleftheria Baltagianni, Maria-Anna Bazmpani, Paraskevi Exadaktylou, Despoina Parcharidou, Thomas Gossios, Argyrios Doumas, Theodoros Karamitsos, Vassilios Vassilikos, Georgios Efthimiadis, Antonios Ziakas, Vasileios Kamperidis

**Affiliations:** 1 Department of Cardiology, AHEPA Hospital, School of Medicine, Aristotle University of Thessaloniki, 54636 Thessaloniki, Greece; georgetzio88@hotmail.com (G.T.); zegkosth@gmail.com (T.Z.); b.elira@hotmail.com (E.B.); mariannabaz@hotmail.gr (M.-A.B.); despoina@parcharidou.eu (D.P.); thomasgossios@hotmail.com (T.G.); tkaramitsos@auth.gr (T.K.); geythymi@auth.gr (G.E.); tonyziakas@hotmail.com (A.Z.); 2 Laboratory of Nuclear Medicine, AHEPA Hospital, School of Medicine, Aristotle University of Thessaloniki, 54636 Thessaloniki, Greece; voulaexadaktylou@hotmail.com (P.E.); argirisdumas@icloud.com (A.D.); 3 Department of Cardiology, Ippokrateio Hospital, School of Medicine, Aristotle University of Thessaloniki, 54642 Thessaloniki, Greece; vvassil@auth.gr

**Keywords:** ATTR, cardiac amyloidosis, cardiomyopathy, imaging, diagnosis, transthyretin, echocardiography, cardiac magnetic resonance, DPD scintigraphy

## Abstract

Amyloidosis is an infiltrative disease that may cause cardiomyopathy if the precursor protein that misfolds and forms the amyloid is transthyretic or plasma abnormal light chains. Transthyretin amyloid cardiomyopathy has to be diagnosed timely and accurately since there are specific treatment options to support the patients. Multimodality imaging including electrocardiography, echocardiography with strain imaging and cardiac magnetic resonance applying late gadolinium enhancement imaging, native T1 mapping and extracellular volume, raise a high suspicion of the disease and bone scintigraphy set the diagnosis even without the need of biopsy. However, the morbidity and mortality remain high and the need for risk stratification and assessment of the response to treatment are of paramount importance. Cardiac imaging biomarkers offer a thoughtful insight into the prognosis of these patients at diagnosis and after treatment. The current narrative review aims to enlighten the use of multimodality cardiac imaging in transthyretic amyloid cardiomyopathy throughout the disease pathogenesis and evolution from diagnosis to prognosis and response to treatment in a personalized manner.

## 1. Introduction

Amyloidosis is a rare protein misfolding disease characterized by a buildup of abnormal amyloid deposits in various organs, leading to progressive organ dysfunction [1]. Commonly affected organs include the heart, kidney, liver, peripheral nerves, and autonomic nervous system. Cardiac Amyloidosis (CA) is the most prevalent cardiac storage disease, marked by the extracellular deposition of amyloid fibrils within the myocardium [2]. These deposits disrupt the normal architecture and function of the heart, leading to restrictive cardiomyopathy and heart failure (HF). Despite the recent advances in diagnostic tools and the growing interest, CA remains an underdiagnosed condition due to its nonspecific clinical presentation and overlap with other cardiac conditions.

CA represents the leading cause of mortality in patients with systemic amyloidosis. Gilstrap et al. estimated the incidence of CA at 17 per 100,000 person-years, and a prevalence of 55 per 100,000 person-years [3]. Even though the International Society of Amyloidosis has identified more than 40 proteins to be capable of forming amyloids, only 10 amyloidogenic proteins accumulate in the myocardium and cause CA [4]. Among these, responsible for over 98% of CA cases are immunoglobulin light chains (AL) and transthyretin amyloidosis (ATTR), either in its hereditary (ATTRv) or acquired (ATTRwt) form [5].

**Figure 1:**
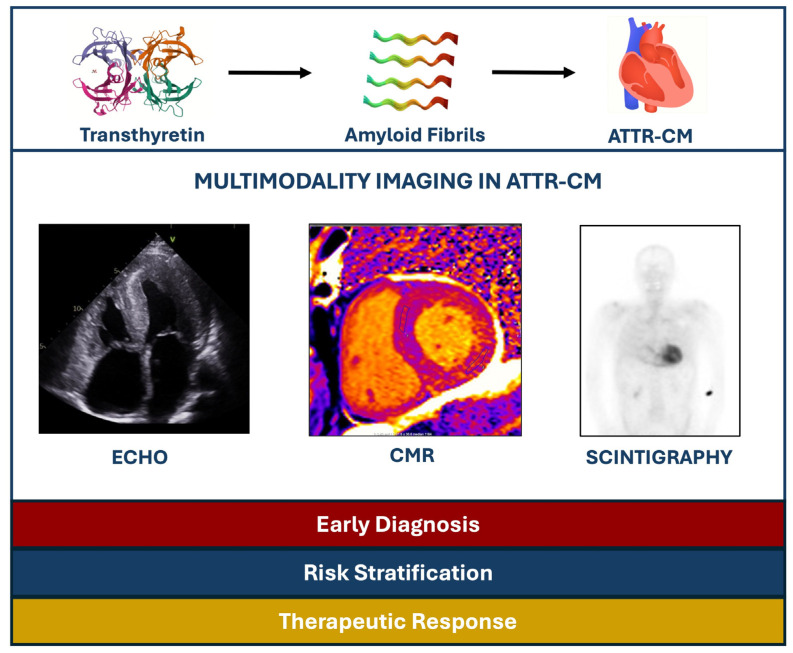
Summary of the role of multimodality imaging in ATTR-CM patients. ATTR-CM: Transthyretin amyloid cardiomyopathy, CMR: Cardiac magnetic resonance, ECHO: Echocardiography.

The aim of this narrative review is to explore the utility of different imaging modalities in ATTR cardiomyopathy (ATTR-CM) from their diagnostic performance and accuracy to their prognostic value in the era of disease-specific therapies (Figure 1).

## 2. Pathophysiology of CA

Amyloidogenesis begins with the destabilization of native protein structures due to genetic mutations, post-translational modifications, or oxidative stress. Misfolded proteins aggregate into toxic oligomers and β-pleated sheet fibrils, which deposit in the myocardium. This deposition disrupts normal tissue architecture, leading to increased myocardial stiffness, impaired relaxation, and restrictive hemodynamic [6].

AL is the most common subtype of CA [7]. In AL, free light chains (particularly λ light chains) are prone to proteolysis, producing amyloidogenic fragments that are directly cardiotoxic [8].

In ATTR, destabilization of the transthyretin tetramer, a liver-derived plasma protein, leads to the formation of monomers, which misfold, aggregate, and form amyloid fibrils. Transthyretin normally circulates as a stable tetramer, transporting thyroxine and retinol-binding protein [9]. Both ATTRwt and ATTRv contribute to progressive myocardial damage, with the latter influenced by specific mutations that affect fibril composition and organ tropism [10]. Specifically:

- ATTRwt is a non-hereditary form primarily affecting elderly men [11]. ATTRwt usually manifests as HF with preserved ejection fraction (HFpEF) and is frequently preceded by systemic symptoms such as bilateral carpal tunnel syndrome or lumbar spinal stenosis [12].
- ATTRv is caused by autosomal dominant mutations in the transthyretin gene, located on chromosome 18, which encodes amyloidogenic variants with increased propensity to misfold.

Over 120 pathogenic mutations have been identified, including Val122Ile, Thr60Ala, and Val30Met [13]. These mutations lead to diverse clinical presentations, ranging from peripheral and autonomic neuropathy to cardiomyopathy. The disease onset varies depending on the mutation but commonly occurs after age 40. For example, Val122Ile is highly prevalent among African Americans (4%) [14] and predisposes to late-onset CA, often indistinguishable from ATTRwt, while Thr60Ala and other mutations may present with mixed neuropathic and cardiac phenotypes [15].

The pathophysiological impact of amyloid deposits is multifactorial. Mechanically, amyloid fibrils disrupt myocardial compliance, leading to diastolic dysfunction and restrictive cardiomyopathy [16]. Functionally, amyloid deposition in the atria contributes to atrial arrhythmias, while involvement of the conduction system predisposes patients to atrioventricular block and bradyarrhythmias [17]. Cytotoxic oligomers also cause direct membrane injury, interfering with calcium handling and mitochondrial function, further impairing contractility. Progressive ventricular wall thickening, often misinterpreted as hypertrophy, is accompanied by low electrocardiographic (ECG) voltage and elevated cardiac biomarkers such as NT-proBNP and troponin, reflecting the combined effects of myocardial stress and damage [6,18].

## 3. Clinical Suspicion of ATTR-CM

AL often manifests with multisystem involvement, including unexplained HF, nephrotic syndrome, autonomic or peripheral neuropathy, and hepatomegaly. ATTR-CM progresses more insidiously, primarily affects older men and often presents as unexplained HFpEF, accompanied by signs of restrictive cardiomyopathy including dyspnea on exertion and lower extremity oedema due to elevated right-sided filling pressures as well as pleural effusions and conspicuous absence of ischemic symptomatology [19,20]. To lend more support several studies identified that over 10% of patients hospitalized for HFpEF suffered from ATTRwt [5]. However, there are also other ideal populations for ATTR-CM screening in the presence of red flags. For example, approximately 16% of patients with severe aortic stenosis (AS) undergoing transcatheter valve replacement had coexisting evidence of ATTR-CM [21]. Accordingly, previous studies identified 9% prevalence of the disease among individuals with hypertrophic cardiomyopathy (HCM) over 40 years old, with at least one red flag for the diagnosis of ATTRwt.

Common clinical red flags for ATTR-CM include:

- Unexplained HFpEF,
- ECG findings of low voltage despite left ventricular wall thickening,
- A ECG pseudo-infarction pattern, and
- Recurrent hospitalizations for HF despite optimized therapy [22].

Fatigue, progressive dyspnea, arrhythmias such as atrial fibrillation especially if it is slow, atrioventricular blocks, and resistance or intolerance to standard heart failure therapies may also raise suspicion for ATTR-CM [19].

Extracardiac red flags include:

- Autonomic or peripheral neuropathy;
- Carpal tunnel syndrome, especially if it is bilateral;
- Lumbar spine stenosis;
- Biceps tendon rupture.

The extracardiac red flags can precede cardiac symptoms by years [23]. Patients may also have elevated cardiac biomarkers that appear disproportionate to the degree of heart failure. Recognizing these red flags in advance is essential to initiate diagnostic evaluation and improve the prognosis. Diagnostic ECG, echocardiographic and CMR hallmarks of ATTR-CM are shown in Table 1.

## 4. Diagnostic Algorithm of ATTR-CM

**Figure 2:**
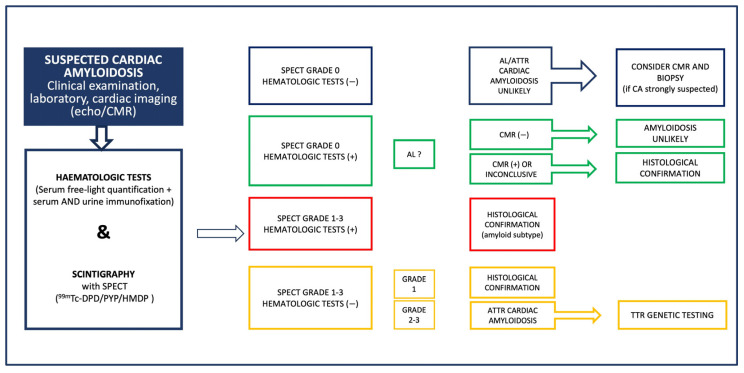
Diagnostic algorithm for cardiac amyloidosis as proposed in the position statement of the European Society of Cardiology Working group on Myocardial and Pericardial diseases for the diagnosis and treatment of cardiac amyloidosis [2]. AL: light-chain amyloidosis, ATTR: transthyretin amyloidosis, CA: cardiac amyloidosis, CMR: cardiac magnetic resonance, ECHO: echocardiography, SPECT: single-photon emission computed tomography, TTR: transthyretin, 99mTc-DPD: 99m technetium 3,3-diphosphono-1,2-propanodicarboxylic acid, 99mTc-HMDP: 99m technetium hydroxymethylene diphosphonate, and 99mTc-PYP: 99m technetium pyrophosphate.

The diagnostic algorithm begins with a high index of suspicion based on clinical, electrocardiographic and echocardiographic features or other imaging red flags provided by cardiac magnetic resonance (CMR) [24]. Of note, CMR alone is not diagnostic for ATTR-CM; however, it can provide significant diagnostic information especially for the differential diagnosis of CA from other cardiomyopathies and infiltrative diseases. Myocardial scintigraphy with bone avid tracers; 99m technetium 3,3-diphosphono-1,2-propanodicarboxylic acid (99mTc-DPD), 99m technetium hydroxymethylene diphosphonate (99mTc-HMDP) and 99m technetium pyrophosphate (99mTc-PYP) is then performed with high diagnostic accuracy [25]. Concomitantly, serum-free light-chain concentration and serum and urine immunofixation electrophoresis are assessed to rule out AL-CA. Myocardial scintigraphy with bone avid tracers is diagnostic of ATTR-CM if there is grade 2 to 3 cardiac uptakes when combined with negative tests for monoclonal light-chain protein with 100% positive predictive value. If ATTR-CM is identified, then genetic sequencing of the transthyretin gene is required to define ATTRv versus ATTRwt disease [26]. The diagnostic algorithm for CA as proposed by the position statement of the European Society of Cardiology for the diagnosis and treatment of CA is shown in Figure 2.

In rare situations, endomyocardial biopsy may still be necessary to establish the diagnosis in the case of negative or equivocal scintigraphy despite a high clinical suspicion, such as very mild disease and specific ATTRv mutations associated with false negative bone scintigraphy, or in the case of plasma cell dyscrasia evidence, irrespective of the positivity of the scintigraphy, to confirm the diagnosis of AL-CA [2].

## 5. Electrocardiographic Features

**Figure 3:**
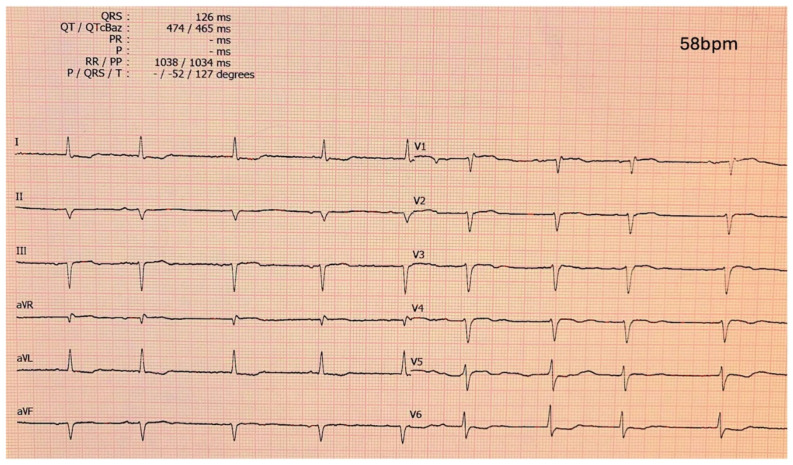
Electrocardiogram of a ATTR-CM patient showing a pseudo-infarction pattern in anterior and inferior leads, low-voltage QRS complexes and atrial fibrillation with slow ventricular response. ATTR-CM: transthyretin amyloid cardiomyopathy.

The 12-lead ECG remains a critical diagnostic tool in CA [27]. ECG findings vary between AL-CA and ATTR-CM, reflecting the differences in the pathophysiology of the disease as well as cardiac involvement. Low QRS voltage, in contradiction to the observed left ventricular hypertrophy (LVH), is a hallmark feature in CA and are observed more frequently in AL-CA (60%) compared to ATTRwt, and ATTRv (25%) [28]. This discrepancy is attributed to the greater cardiomyocyte toxicity of light chains in AL-CA, which exacerbates myocardial infiltration regardless of left ventricular wall thickness [29]. The pseudo-infarction pattern, characterized by Q waves ≥ 1 mV in at least two contiguous leads without a history of ischemic heart disease, is seen in approximately 50–70% of CA patients [30]. Localization of the pseudo-infarction pattern differs; anterior leads are more commonly involved in AL-CA, while inferior leads are more frequently indicative of ATTR-CM. Specific transthyretin mutations, like V122I, may influence ECG characteristics, including an increased prevalence of low QRS voltage (56.7%) and first-degree atrioventricular block. These characteristics underline the importance of ECG in raising the suspicion and ultimately diagnosing CA. A characteristic ECG of a patient with ATTR-CM is shown in Figure 3.

## 6. Echocardiography

### 6.1. Echocardiography for Diagnosis

#### Conventional Echocardiography

Echocardiography is a highly accessible, safe, and cost-effective imaging modality for the assessment of CA [31]. Increased left ventricular wall thickness due to amyloid infiltration is the most prominent echocardiographic finding in CA [32]. LVH can be mild in the early stages of the disease, while normal ventricular wall thickness, should not suffice to rule out CA [33,34]. Current diagnostic algorithms require, along with the presence of cardiac and/or extra-cardiac amyloidosis red flags, LVH ≥ 12 mm to pursue the diagnostic screening for CA [2]. Differentiating CA from other causes of LVH (AS, hypertensive heart disease, HCM) can be challenging since echocardiographic findings between these conditions may overlap, and therefore, clinical awareness is warranted [34].

Left ventricular (LV) cavity in CA is most commonly non-dilated, leading to decreased ventricular volumes [34,35]. In recent reports, the LVH pattern is asymmetrical in ATTR (70% septal sigmoid), whereas hypertrophy in AL-CA typically appears concentric and symmetric [36,37]. Although rare, dynamic LV outflow track obstruction can be seen in cases with asymmetric septal LVH, confounding with obstructive HCM [38].

Except for the LV, amyloid infiltration can be noted in other locations, including the right ventricle (RV), the atria and interatrial septum, the valves, and the pericardium [39]. RV is chronically infiltrated and thickened after LV and RV free wall thickness > 5 mm with decreased RV function can strongly suggest CA [40,41]. Biatrial hypertrophy and enlargement is a result of amyloid deposition in conjunction with elevated ventricular filling pressures due to impaired ventricular relaxation. When the interatrial septum is affected, values greater than 6 mm have 100% specificity for the diagnosis of CA [41]. Valve thickening is another clue for CA. Aortic valve infiltration plays an important role in the pathogenesis and progression of AS. Up to 30% of the low-flow low-gradient AS population may have CA [42]. Atrioventricular valves can be also thickened, leading to regurgitant physiology [39]. Pericardial effusion is present in almost half of CA patients, most frequently in a small amount and a circumferential pattern [34,43].

Systolic function as measured by LV ejection fraction (LVEF) is usually preserved and CA is considered a neglected cause of HFpEF [44]. A decline in EF is observed later during disease progression to advanced stages [43]. A study, however, found EF < 50% in 36% of ATTRwt patients indicating the delay of CA diagnosis [45]. On the other hand, RV systolic dysfunction is common in the early stages of ATTR [46]. RV fractional shortening and tricuspid annular plane systolic excursion are lower among CA patients compared to patients with other causes of LVH [47,48].

Diastolic dysfunction in CA is more pronounced compared to other causes of LVH and tends to worsen with disease progression [34]. Grade 1 diastolic dysfunction is not uncommon in the early stages of the disease [38]. At the time of diagnosis, though, most patients demonstrate grade 2 or higher diastolic dysfunction [49]. Normal E wave, small A wave, high E/A ratio, rapid deceleration time on diastolic mitral inflow (restrictive pattern), and progressive decline in the pulmonary S wave are echocardiographic markers of advanced disease. Tissue doppler imaging shows significantly decreased peak early mitral and tricuspid diastolic velocities (e′), with a high E/A ratio that reflects elevated filling pressures [50]. In ATTR-CM, diastolic dysfunction typically progresses during the disease course, but restrictive physiology especially in AL-CA may occur even before overt LVH [51].

**Figure 4:**
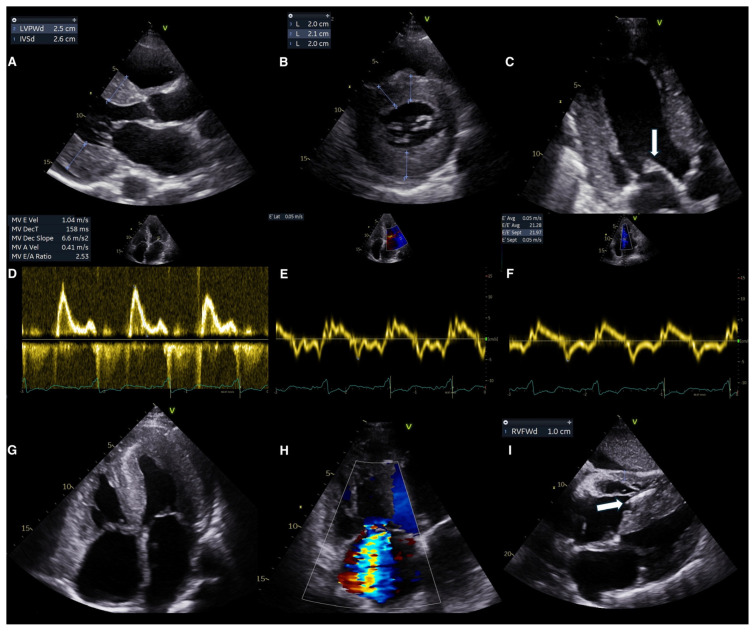
Conventional echocardiographic red flags in ATTR-CM. (**A**,**B**) Concentric hypertrophy of the left ventricle demonstrated at parasternal long axis view (**A**) and short axis view (**B**). (**C**) Thickened mitral valve leaflets (arrow). (**D**–**F**) Grade III diastolic dysfunction demonstrated by (**D**) Transmitral flow (**E**) tissue doppler of the lateral wall and (**F**) tissue doppler of the intraventricular septum. (**G**) Four chamber view demonstrating biatrial enlargement, interatrial septum thickening, right ventricular free wall hypertrophy and pericardial effusion. (**H**) Color doppler imaging demonstrating the significant tricuspid regurgitation. (**I**) Subcostal view demonstrating hypertrophy of the right ventricle and the thickened interatrial and interventricular septum, visible pacemaker lead in the right ventricle (arrow). ATTR-CM: transthyretin amyloid cardiomyopathy, IVSd: interventricular septum diameter, LVPWd: left ventricular posterior wall diameter, MV: mitral valve, MV DecT: mitral valve deceleration time, and RVFWd: right ventricular free wall diameter.

Lastly, the reported granular or sparkling myocardial texture in CA, seen in harmonic imaging, may characterize other conditions as well. Therefore, increased echogenicity may be suggestive but not pathognomonic of CA [38,52]. Conventional echocardiographic red flags in CA are shown in Figure 4.

### 6.2. Advanced Echocardiography

**Figure 5:**
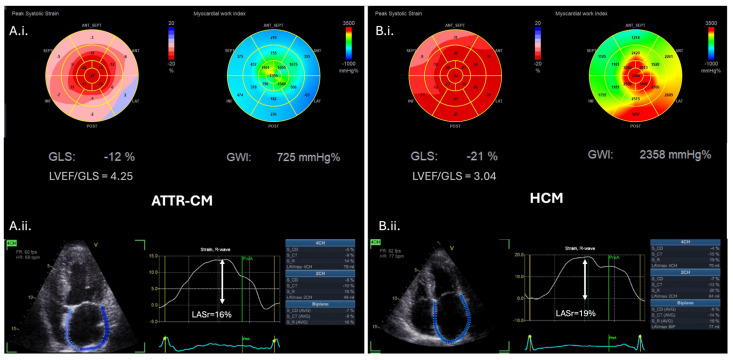
Advanced echocardiographic red flags in ATTR-CM (**A.i.**,**A.ii.**) versus hypertrophic cardiomyopathy (HCM) patients (**B.i.**,**B.ii.**). (**A.i.**) Bull’s eye plots of left ventricular global longitudinal strain (GLS) and myocardial work index (MWI) in an ATTR-CM individual demonstrating an apical sparing pattern versus (**B.i.**) the respective Bull’s eye plots of a HCM individual demonstrating reduced left ventricular GLS and MWI of the hypertrophic interventricular septum. (**A.i.**) and (**B.i.**) LVEF/GLS ratio is higher in CA compared to HCM (cut-off value LVEF/GLS ratio > 4.1 to diagnose CA with 89% sensitivity and 91% specificity) [55]. (**A.ii.**) Left atrial strain (LAS) analysis of the ATTR-CM patient versus (**B.ii.**) the respective LAS analysis of the HCM patient, indicating the lower peak values of left atrial reservoir (LASr) and contractile strain characterizing ATTR-CM. ATTR-CM: transthyretin amyloid cardiomyopathy, CA: cardiac amyloidosis, GLS: global longitudinal strain, HCM: hypertrophic cardiomyopathy, LAS: left atrial strain, LASr: left atrial reservoir strain, LVEF: left ventricular ejection fraction, and MWI: myocardial work index.

LV strain imaging measured by speckle tracking echocardiography is widely considered a more robust technique than conventional tissue doppler imaging for the assessment of subtle changes in myocardial function [53]. In CA, longitudinal function is affected before radial function, and therefore, reduction in the LV global longitudinal strain (GLS) can detect subclinical systolic dysfunction of the LV, even when LVEF is still preserved, and HF symptoms may not be overt [34,37,39]. GLS values are greater in the apical segments and severely impaired in the basal to middle segments, creating a distinct “apical sparing” pattern on the bull’s eye plot [31]. This pattern, however, can be also seen in other infiltrative cardiomyopathies, and although highly suggestive, it is not specific to CA. LVEF/GLS ratio has been reported as a better screening tool for CA among a heterogenous group of patients with LVH, demonstrating 75% sensitivity and 66% specificity when LVEF/GLS > 4.95, and 93% sensitivity and 38% specificity for values > 4.1 [54]. Another study that specifically compared CA to HCM and hypertensive individuals showed that LVEF/GLS > 4.1 was the best predicting parameter of CA diagnosis in this population with 89% sensitivity and 91% specificity (Figure 5) [55].

Moreover, a systolic septal longitudinal base-to-apex strain ratio > 2.1 combined with a shortened diastolic deceleration time of early filling <200 ms is another echocardiographic marker that aids in differentiating CA from other causes of concentric LVH [56]. In general, absolutes values of apical strain are lower in ATTR-CM than in AL-CA, but distinguishing CA subtype based on strain measurements alone cannot be justified [35]. With respect to RV speckle tracking echocardiography, values of free wall RV longitudinal strain significantly deteriorate during follow-up [40]. A RV apical sparing pattern is common in CA and RV apical/(basal + middle) ratio > 0.8 may perform well in differentiating AL-CA from ATTR-CM [57].

Left atrial (LA) strain analysis by STE is an important tool for the evaluation of LA function in patients with CA, detecting volumetric changes before traditional echocardiography [53]. Atrial contraction may be absent in up to 22% of CA patients in sinus rhythm, and this electromechanical dissociation increases the thromboembolic risk [58]. Accordingly, all the three phases of atrial strain, including reservoir, conduit, and contraction function are more impaired than matched healthy controls, hypertensive or HCM individuals (Figure 5) [34,59]. Importantly, impaired LA strain parameters represent a significant early marker for the differential diagnosis between CA and HCM [60]. Between CA subtypes, LA reservoir, and LA contraction stain are more severely affected in ATTRwt than ATTRv and AL-CA [61]. Of note, LA mechanical dispersion is a novel marker of intra-atrial dyssynchrony implicated in LA myopathy that can differentiate ATTR from AL-CA with 80% specificity, even in sinus rhythm or when LVH is mild [62].

Myocardial work is novel echocardiographic tool that incorporates LV pressure into the strain measurement, providing a load-independent evaluation of systolic LV function [63]. Myocardial work parameters have been evaluated for their increased diagnostic and prognostic value in multiple clinical settings, including ischemic heart disease, valvular heart disease and cardiomyopathies considering the challenges in the diagnostic process, the differential diagnosis of a hypertrophied myocardium and the lack of advanced risk stratification algorithms [64]. Global myocardial work index (GWI) and global constructive work are lower in ATTR-CM than in other hypertrophic phenocopies (Figure 5) performing better diagnostically than GLS [65]. Moreover, contrary to less than half of HCM patients, the majority of ATTR-CM individuals demonstrates impaired GWI in combination with low systemic vascular resistance (arterial–ventricular coupling parameters) [66]. With respect to CA subtypes, it has been reported that AL-CA patients had significantly lower global myocardial work efficiency compared to ATTRwt patients, and values < 86.5% could differentiate AL-CA from ATTR-CM with 80% sensitivity and 66.7% specificity [67]. Interestingly, another study demonstrated that GWI was a valuable marker to distinguish ATTRwt with AS from lone aortic AS [68].

### 6.3. Echocardiography for Prognosis

Numerous echocardiographic parameters bear prognostic implications for patients with CA. Early reports indicated that increased LVEF wall thickening, low EF, decreased fractional shortening, and markers of diastolic dysfunction were associated with poorer prognosis. LA dilation and increased E/A ratio with shorter deceleration time strongly correlated with advanced HF and mortality in CA [69]. Myocardial contraction fraction, defined as stroke volume over myocardial volume, is another index of myocardial function, superior to LVEF in predicting mortality in both ATTR-CM and AL-CA [35]. Evaluation of RV systolic dysfunction has been also implicated in the risk stratification of patients with CA. Tricuspid annular plane systolic excursion < 14 mm is considered an independent predictor of acute HF, death, and heart transplantation [70]. Although rare, RV dilation may occur in advanced disease and confers a poor prognosis [71]. Pericardial effusion has been reported as a prognostic marker, irrespective of CA subtype, while ATTR patients with concomitant severe aortic stenosis have a trend to worse prognosis (median survival 23 vs. 55 months) [72]. Last, the degree of mitral and tricuspid regurgitation may negatively affect survival [73].

In recent publications, myocardial strain conferred incremental prognostic value for CA [74]. GLS has been established as an independent predictor of mortality in CA [50]. To lend more support, apical LS > −14.5% has been reported to independently predict cardiovascular events [75]. With respect to RV, impaired free wall longitudinal strain at baseline has been linked to adverse outcomes [76]. Worse short- and long-term survival during follow-up has been reported in CA patients with RV free wall strain <16% [77]. With respect to LA function, the reservoir strain has been identified as an independent predictor of events performing even better than GLS and RV strain [78]. Myocardial work indices also seem to have additive value for outcome prediction in CA with various reported cut-off values. Recently, a study reported that GWI and global wasted work were associated with mortality and HF hospitalization in ATTRwt on top of RV and LA deformation parameters [79].

### 6.4. Echocardiography for Monitoring the Response to Treatment

Tafamidis, a transthyretin tetramer stabilizer that improved prognosis in ATTR patients with HF, is currently the only approved treatment for ATTR-CM, either wild-type or hereditary [80,81]. Monitoring disease progression in ATTR-CM is challenging and lacks validated criteria. Several modalities have been utilized to achieve an effective monitoring of treatment response. For example, cardiopulmonary exercise testing’s objective parameters such as maximal oxygen consumption, ventilator efficiency and exercise capacity have been studied for their value in predicting the disease’s outcomes, therapeutic response and tailored therapeutic decisions with promising results [82]. An expert consensus proposed measurement tools for detecting ATTR-CM progression in treated patients across three clinical domains (clinical, biomarkers, and ECG/imaging). According to this consensus, increased LV wall thickness > 2 mm, an increase in diastolic dysfunction grade or change in systolic measurements (≥5% decreases in LVEF or a ≥5 mL decrease in LV stroke volume or ≥1% increase in LV GLS) are echocardiographic markers indicative of disease progression that should be performed every 6 to 12 months to assess response to treatment [83]. In a post hoc analysis of the ATTR-ACT trial, tafamidis, compared to placebo, attenuated the decline of systolic and diastolic function (LVEF, LV stroke volume, LV GLS, E/E’) in patients with ATTR-CM [84]. Real-world studies have also assessed the role of echocardiography in monitoring disease progression and response to treatment. A study of 41 tafamidis-treated patients with ATTR-CM did not report significant changes in various representative echocardiographic markers (LVEF, LV mass, E/e′, LA volume index, GLS, relative apical sparing) after tafamidis administration, indicating its potential to halt the disease progression [85]. Giblin et al. demonstrated that treatment with tafamidis resulted in less deterioration in GLS and myocardial work-derived parameters over 12 months [86]. Rettl et al. evaluated the impact of tafamidis on myocardial strain in ATTR-CM and found that treatment with tafamidis delays the worsening of LV GLS and LA reservoir strain compared to treatment-naïve patients, resulting in clinical benefits [87]. Lastly, a recent metanalysis confirmed that tafamidis significantly slowed the deterioration of GLS [88].

## 7. Cardiac Magnetic Resonance

### 7.1. CMR for Diagnosis

Cardiovascular magnetic resonance, with its unique ability to provide detailed anatomical and morphological information along with tissue characterization, holds great significance in the non-invasive diagnosis of CA. In the proposed diagnostic algorithm provided by the European Society of Cardiology Working Group on Myocardial and Pericardial diseases, CMR should be considered for patients with negative hematologic tests and grade 0 scintigraphy, if suspicion of CA persists, and in patients with positive hematologic tests and grade 0 scintigraphy, to detect cardiac involvement in AL [2].

**Figure 6:**
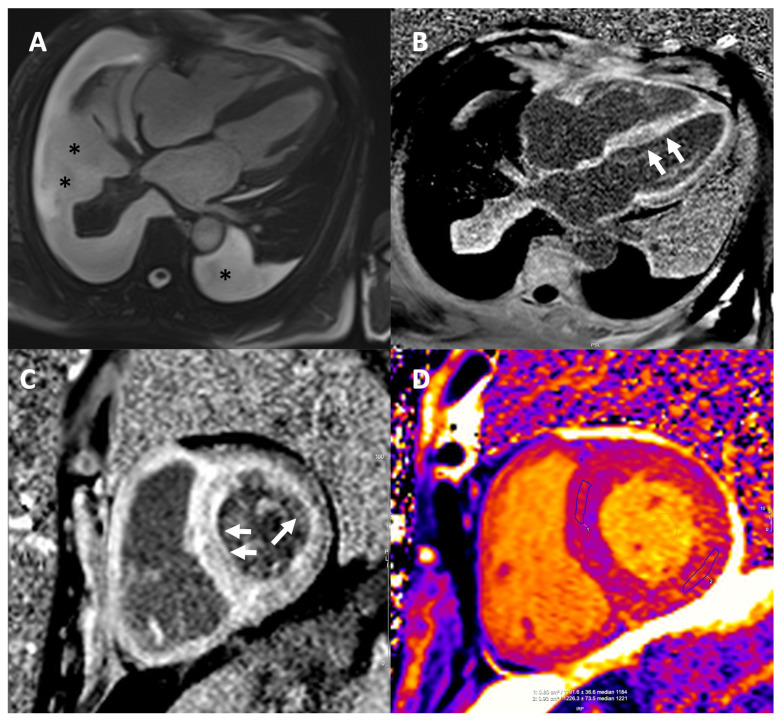
CMR in a patient with CA. (**A**) End-diastolic frame from horizontal long axis cine demonstrating left ventricular hypertrophy (LVH) and pericardial and pleural effusions (black asterisks *). (**B**,**C**) Late gadolinium images in the horizontal long axis (**B**) and mid short axis (**C**) showing the characteristic diffuse subendocardial/transmural myocardial enhancement (white arrows) and dark blood pool. Infiltration of the right ventricle, atrial walls and papillary muscles is also seen. (**D**) Modified Look Locker Inversion Recovery Native T1 Mapping showing markedly elevated T1 times in all myocardial segments. Extracellular volume (ECV) was also significantly elevated (ECV of 52%, normal values 22–27%). CA: cardiac amyloidosis, ECV: extracellular volume, and LVH: left ventricular hypertrophy.

CMR demonstrates characteristic findings in patients with CA (Figure 6) and thus, aids differentiation from other causes of LVH [89]. Specifically, CMR is important for the differential diagnosis of hypertrophic phenocopies with its ability to provide multiparametric tissue characterization. On this basis, the utilization of T1 and T2 mapping has made feasible the quantitative assessment of myocardial tissue characterization providing an important tool for the diagnostic process of hypertrophic phenotypes and the monitoring of the therapeutic response especially in the context of disease-modifying drugs in infiltrative cardiomyopathies [90]. Although echocardiography is the first-line diagnostic imaging modality, the key advantage of CMR is tissue characterization using late gadolinium enhancement (LGE) imaging which requires intravenous administration of gadolinium-based contrast agents. A characteristic pattern of global subendocardial or transmural enhancement along with abnormal gadolinium kinetics and difficulty in myocardial nulling have been identified as hallmarks for CA with high sensitivity and specificity reported across studies [91,92,93]. This distinct LGE pattern has been associated with the expansion of interstitial space attributed to amyloid infiltration, as shown by Maceira et al. who compared imaging findings with cardiac histology, which is the gold standard [91].

The expanding implementation of parametric mapping techniques and measurement of extracellular volume (ECV) have redefined the importance of CMR in detecting cardiac involvement in amyloidosis and differentiating from other diseases such as Anderson–Fabry and aortic stenosis [89]. While LGE is of paramount importance for diagnosis of CA, renal impairment is frequent in patients with amyloidosis and administration of gadolinium-based contrast agents is problematic when glomerular filtration rate is below 30 mL/min/1.73 m^2^.

Native T1 mapping is a non-contrast CMR technique that provides quantitative assessment of myocardial amyloid infiltration. T1 mapping is affected by both intracellular as well as extracellular/interstitial processes [94]. It has been proven to be substantially elevated in patients with definite cardiac involvement as well as patients with possible cardiac involvement or no cardiac involvement based on echocardiographic findings and serum biomarkers [95]. In a large cohort including patients with both AL-CA and ATTR-CM, native T1 mapping has been demonstrated to have an excellent diagnostic accuracy in patients with clinically suspected cardiac amyloidosis and a native T1 > 1164 ms yielded a positive predictive value of 98% [96]. Furthermore, the administration of gadolinium-based contrast agents enables measurement of post-contrast T1 values and ECV. Deposition of amyloid occurs only in the interstitial space, between myocardial cells. Since ECV reflects the expansion of interstitial space, it is substantially raised in patients with cardiac amyloidosis, even in patients with early-stage disease, both in AL and ATTR-CM [97]. An ECV cut-off value of 0.469 yielded a sensitivity of 93% and a specificity of 82% for diagnosis of ATTR-CM [98]. The normal range for ECV is between 20% and 26% and an ECV value over 40% is highly suggestive of CA [80,99]. Pan et al., in their systematic review and meta-analysis, highlight the comparable diagnostic value of native T1 mapping and ECV to LGE for detecting CA and in fact, the superior diagnostic performance of ECV compared to LGE [100]. Furthermore, ECV has been reported to be elevated even when no LGE is detected, rendering ECV an early marker of disease [101]. CMR may also have some value in differentiation between AL amyloidosis and ATTR-CM. A more diffuse and transmural LGE pattern is more common in ATTR-CM, whereas native T1 values are higher in AL amyloidosis and ECV higher in ATTR-CM [102]. Dungu et al. investigated the application of QALE score, which assessed the presence and extend of LGE in three LV short axis slices and the right ventricle and demonstrated that a QALE score ≥ 13 yielded a sensitivity of 87% and a specificity of 96% for ATTR-CM [103]. However, it should be kept in mind that there is significant overlap between the two subtypes of CA and CMR alone cannot reliably distinguish between AL-CA and ATTR-CM.

Taking these findings into consideration, T1 mapping and ECV measurement have been incorporated into clinical routine along with the established LGE imaging.

### 7.2. CMR for Prognosis

Several CMR markers have gained acceptance for their prognostic value in cardiac amyloidosis. The pattern and extend of LGE, reflecting the degree of myocardial involvement, have been identified as a significant prognostic marker. In particular, patients with transmural LGE have the worst prognosis, followed by patients with subendocardial LGE, both in the case of AL-CA and ATTR-CM [102]. In the meta-analysis by Pan et al., there was no significant difference in the diagnostic performance of LGE and T1 mapping. Nonetheless, the prognostic performance of ECV was significantly higher compared to both LGE and T1 [100].

### 7.3. CMR for Monitoring the Response to Treatment

Apart from prognostication, another emerging role of CMR is tracking changes in myocardial amyloid burden as a surrogate marker for response to treatment. Regression of LGE and reduction in native T1 and ECV following response to chemotherapy have been documented in patients with AL-CA [101]. ECV is also useful in monitoring the effects of treatment with tafamidis in ATTR-CM [104]. Since novel therapies are currently investigated, CMR evidence of disease regression could serve a clinical-trial endpoint and guide therapeutic management.

## 8. Nuclear Medicine

### 8.1. Nuclear Medicine for Diagnosis

Identifying whether amyloid is AL or ATTR is critically important as it is inextricably linked to patients’ clinical management and prognosis. Nuclear medicine plays a pivotal role in the diagnosis of ATTR-CM. CA radionuclide imaging (CARI) with bone seeking radiotracers (99mTc-PYP, 99mTc-DPD and 99mTc-HMDP) can confirm the diagnosis of ATTR-CM in cases where serum and urine tests for AL are negative, thereby obviating the need for a cardiac biopsy [105]. In fact, at the latest World Heart Federation Consensus on ATTR-CM [106] it was highlighted that scintigraphy is the only diagnostic method that can establish the diagnosis reducing the need of a biopsy. The mechanism of myocardial uptake of bone seeking radiopharmaceuticals in CA has not been fully illuminated. It is possibly attributed to the binding of the tracer to microcalcifications which, as indicated by various studies, are more abundant in ATTR-CM compared to AL-CA [107].

The radiotracer 99mTc-DPD demonstrates superiority over the 99mTc-HMDP in verifying CA cases [108], since the identification of soft tissue uptake, consistent with amyloid deposition, is more prevalent with it, while the phenomenon of the persistent cardiac blood pool, necessitating delayed imaging, is more commonly occurring with the radiotracer 99mTc-PYP [109]. In Europe, nowadays, the radiotracer 99mTc-DPD is mainly used. In the United States, the radiotracer 99mTc-PYP is used and the 99mTc-HMDP is considered a suitable alternative, since the 99mTc-DPD is not yet approved by the Food and Drug Administration.

Details for the acquisition protocols of CARI are comprehensively analyzed in the most recent guidelines. Parameters for improving quality of CARI on the basis of indications, patient experience, workflow, instrumentation and scintigraphy interpretation were recently published by the American Society of Nuclear Cardiology [99,110].

**Figure 7:**
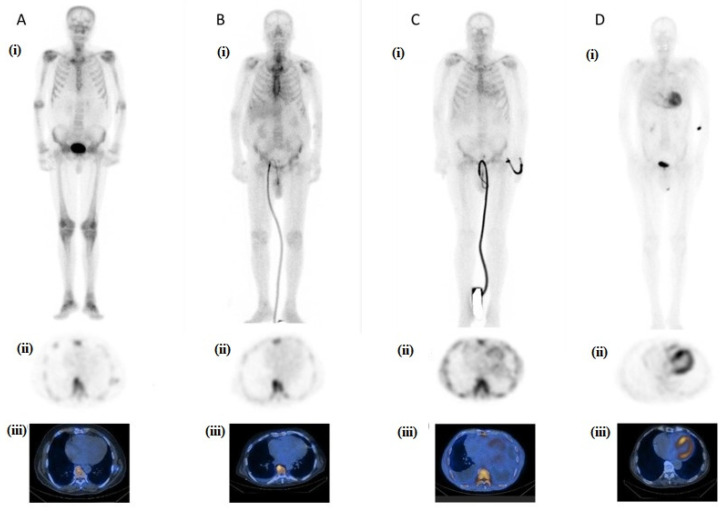
Perugini Score assessment: Grades—(**A**) Grade 0, no cardiac uptake; (**B**) Grade 1, cardiac uptake less than the bone uptake; (**C**) Grade 2, mild cardiac uptake equal to bone uptake; (**D**) Grade 3, intense cardiac uptake with faint/absent bone visualization. Type of imaging obtained 2 h post injection of 20 mCi 99mTc-DPD—(**i**) Whole-body images; (**ii**) SPECT images; (**iii**) SPECT/CT images. 99mTc-DPD: 99m technetium 3,3-diphosphono-1,2-propanodicarboxylic acid, and SPECT/CT: single-photon emission tomography with computed tomography.

Assessment of radiopharmaceutical myocardial uptake in planar imaging is performed qualitative by a visual assessment score, the Perugini score, and semi-quantitatively using the heart to contralateral ratio (H/CL). The Perugini score compares cardiac uptake to the surrounding bones (0: no cardiac uptake, 1: cardiac uptake less than the bones, 2: mild cardiac uptake equal to bone uptake, and 3: intense cardiac uptake with faint/absent bone visualization (Figure 7) [111].

Grades 2 and 3 set the diagnosis of ATTR-CM as long as serum and urine tests exclude the presence of clonal dyscrasia. Grade 0 rules out ATTR-CM while grade 1 could refer to AL, early onset of ATTR-CM or a false positive result [25]. The semi-quantitate method in planar imaging is based on the ratio of cardiac mean counts to the contralateral chest mean counts (H/CL). One-hour post injection, H/CL > 1.3 or 3 h post injection, H/CL ≥ 1.5 is deemed positive for ATTR-CM [99].

Single-photon emission computed tomography (SPECT) with computed tomography reduces the likelihood of false positive results attributed to blood pool. Various other markers are being explored in SPECT imaging as well as in positron emission tomography (PET)/CT imaging, such as SUV, % injected dose, cardiac amyloid activity [112].

PET/CT has not yet been utilized for the diagnostic workup of ATTR-CM. There is growing research though in the potential use of 18F-NaF, a PET bone radiotracer, for the diagnosis of CA, but further studies are warranted [113].

### 8.2. Nuclear Medicine for Prognosis

Regarding the prognostic role of CARI for patients with ATTR-CM, Hutt et al., in a large cohort (*n* = 602), stratified patients by Perugini Grade and found no correlation with patients’ survival [114]. On the contrary, Castano et al., in a multicenter study, showed that a H/CL ≥ 1.6 was linked to poorer survival outcomes in patients with ATTR-CM [115]. An interesting finding emerges from the study of Rettl et al., which demonstrated the correlation between 99mTc-DPD quantification (in terms of DPD retention index) and adverse outcomes [116].

### 8.3. Nuclear Medicine for Monitoring the Response to Treatment

The role of CARI evaluating the response to treatment has not been fully clarified yet. According to the American Society of Nuclear Cardiology, performing serial CARI is not recommended for this purpose as literature has not provided sufficient data [99,110]. It is noteworthy, however, that this recommendation relies on an older study by Castano et al., which evaluated a small cohort of patients (*n* = 20) using 99mTc-PYP. The study used only Perugini grade and H/CL ratio, and the majority of the patients did not receive therapy (only two patients received Tafamidis) [117].

However, contemporary studies, have shown promising results regarding the role of CARI. Papathanasiou et al. evaluated the treatment response in 14 patients who received Tafamidis using 99mTc-DPD scan, they estimated the Perugini score, H/CL ratio and SUVmax in comparison with biomarkers and echocardiography and noticed that CARI parameters were reduced in the post therapy follow up, while the rest remained stable [118]. Recently, Vijayakumar et al. published an article regarding the use of 99mTc-PYP in monitoring patients receiving transthyretin stabilization therapy (*n* = 22 Tafamidis, *n* = 1 Diflunisal). They also noted that molecular metrics reduced in the follow up (in terms of visual grading, SUVmax, cardiac amyloid activity and % injected dose) while echocardiographic metrics and biomarkers exhibited stability. The authors suggested that the molecular changes may reflect alterations in fibrils structure rather than regression of the amyloid burden [119].

In our institution, the potential role of a novel index, named “AHEPA Index”, proposed by Doumas et al. in 2022 is explored for the evaluation of the response to treatment in planar imaging. This index is the geometric mean of the ratio: myocardial mean counts to thigh mean counts and the preliminary results of a small sample of patients (*n* = 5) receiving Tafamidis are promising; however, a larger cohort is currently collected [120].

The optimal period to evaluate response to treatment has not been determined yet. Some studies have observed molecular changes as early as the first 6 months from treatment onset [121,122].

With the emergence of new therapies for ATTR-CM, it is crucial to implement the appropriate method and time for evaluating treatment response and it seems that molecular changes provided by CARI appear to be helpful in this regard. Further studies are warranted to reach a reliable conclusion.

## 9. Conclusions

ATTR-CM remains an underdiagnosed condition associated with increased morbidity and mortality. However, advances in novel imaging techniques enable the non-invasive and early diagnosis of the disease, with significant impact on risk stratification and prognosis. In the era of available disease-modifying treatments, the use of specific imaging markers is imperative to establish an accurate disease-monitoring algorithm. Future clinical trials are needed to assess the timely identification of responders among treated patients to guide therapeutic decisions in an individual and cost-effective manner. Multimodality imaging holds the pivotal role for the achievement of the aforementioned goals. Novel imaging parameters are being identified and implemented in everyday clinical practice to effectively complete the reciprocal link between monitoring the disease progression and response to treatment, with the final aim of improving the quality of life and prognosis of ATTR-CM patients.

## Figures and Tables

**Table 1:** Diagnostic hallmarks of ATTR-CM on electrocardiogram, echocardiogram and cardiac magnetic resonance.

ECG	Echocardiography	CMR
Low QRS voltages (more common in AL-CA) Pseudo-Q waves Atrioventricular block Bundle branch block Atrial fibrillation (with slow ventricular response) Slow heart rate	Increased left ventricular wall thickness (symmetric and asymmetric) Thickened valves Aortic stenosis (most commonly low-flow low-gradient) Biatrial enlargement Thickened interatrial septum Right ventricular hypertrophy Pericardial effusion Granular myocardium Advanced diastolic dysfunction (most commonly grade 2 or 3) Reduced left ventricular GLS with an apical sparing pattern Reduced left atrial reservoir strain (irrespective of left atrial volume) Significantly reduced or absence of left atrial contractile strain even in sinus rhythm Reduced myocardial work index with an apical sparing pattern	Increased values of T1 mapping Increased ECV (especially >40%) Abnormal gadolinium kinetics (difficulty in myocardial nulling) Diffuse subendocardial or transmural LGE

ATTR-CM: transthyretin amyloid cardiomyopathy, GLS: global longitudinal strain, ECV: extracellular volume, and LGE: late gadolinium enhancement.

## Data Availability

No new data were created or analyzed in this study.

## Abbreviations

AL	Light-chain amyloidosis
AL-CA	Light-chain cardiac amyloidosis
AS	Aortic stenosis
ATTR	Transthyretin amyloidosis
ATTR-CM	Transthyretin amyloid cardiomyopathy
ATTRv	Hereditary (variant) transthyretin amyloidosis
ATTRwt	Wild-type transthyretin amyloidosis
CA	Cardiac amyloidosis
CARI	Cardiac amyloidosis radionuclide imaging
CMR	Cardiac magnetic resonance
ECG	Electrocardiogram
ECV	Extracellular volume
GLS	Global longitudinal strain
GWI	Global myocardial work index
HCM	Hypertrophic cardiomyopathy
HF	Heart failure
HFpEF	Heart failure with preserved ejection fraction
H/CL	Heart to contralateral ratio
LA	Left atrium
LGE	Late gadolinium enhancement
LV	Left ventricle
LVEF	Left ventricular ejection fraction
LVH	Left ventricular hypertrophy
PET	Positron emission tomography
RV	Right ventricle
SPECT	Single-photon emission computed tomography
99mTc-DPD	99m Technetium 3,3-diphosphono-1,2-propanodicarboxylic acid
99mTc-HMDP	99m Technetium hydroxymethylene diphosphonate
99mTc-PYP	99m Technetium pyrophosphate
